# Supplementary material for: Mechanoradicals in tensed tendon collagen as a source of oxidative stress
Source: Nat Commun. 2020 May 8;11:2315. doi: 10.1038/s41467-020-15567-4 (PMC7210969; doi:10.1038/s41467-020-15567-4)
Supplement: Supplementary file 1 — Supplementary Information [file 41467_2020_15567_MOESM1_ESM.pdf]

Supplementary information for
“Mechanoradicals in tensed tendon collagen as a source of oxidative stress”
(Zapp and Obarska-Kosinska et al)
including supplementary figures and tables.

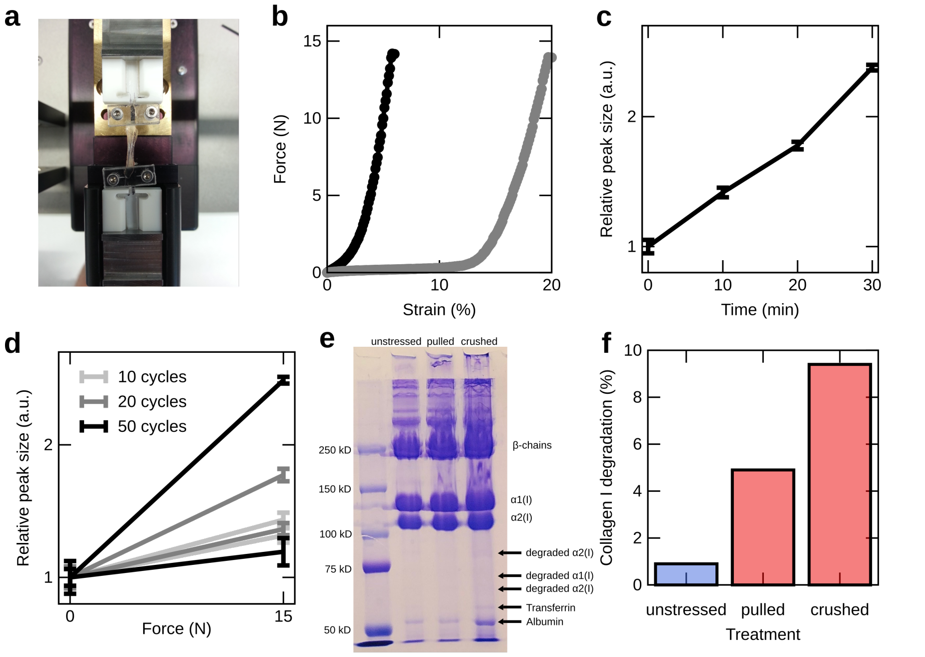

Supplementary Figure 1: **Strain due to the stress of the extensometer.** **a**, LEX810 with custom clams to pull on the rat tail tendon piece. **b**, Stress-strain curve of a 15 N pulling for 25 %hum (black) and 50 %hum (gray) without tendon failure. **c**, Relative EPR peak size recorded at room temperature of a tendon against its pulling time with a load of 200 g against gravity. **d**, Relative EPR peak size for unstressed fascicles (0 N) and fascicles repetitively pulled to a maximum force of 15 N for a different number of cycles shown in different grey tones. Errors show standard deviations calculated from 20 EPR sweeps. **e**, SDS-PAGE of collagen I. Column 1 marker, column 2 unstressed tendon, column 3 tendon pulled with 15 N, column 4 crushed tendon, respectively. **f**, Collagen I degradation in percent of the overall collagen I from densitometry analysis of SDS-PAGE (**e**).

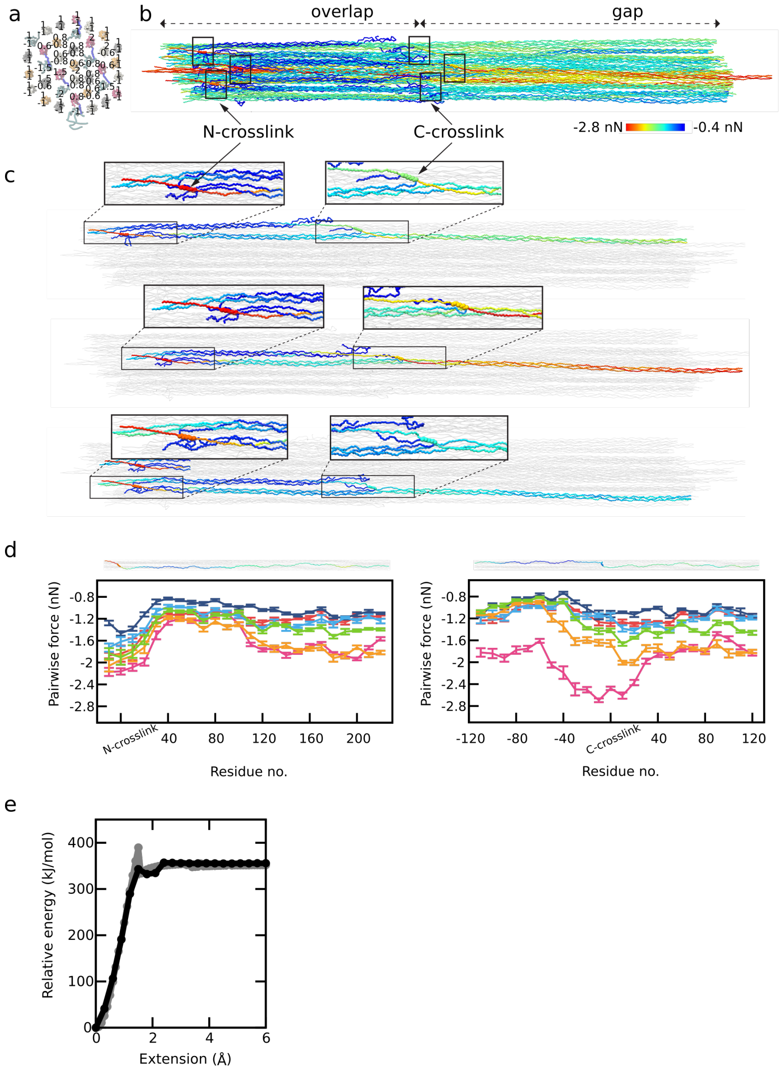

Supplementary Figure 2: **Force propagation within the collagen fibril during shear pulling simulation.** **a**, The pulling forces per chain (in nN) are indicated at the cross-section of the fibril. The positive and negative signs designate the pulling direction outward or inwards of the cross-section plane, respectively. **b**, Random snapshot of the simulation colored according to pairwise force calculated using FDA (blue – low force, red – high force). **c**, Example pairs of overlapping triple helices connected by crosslinks from the snapshot shown in **b**, depicted separately to better visualize forces around the crosslinks. The example pairs are shown in stick representation colored according to forces as in **b**, crosslinks are depicted in sphere representation, whereas the remaining collagen chains as gray ribbons. **d**, Pairwise forces averaged over 10 consecutive residues along the collagen chains connected by crosslinks (left for N-crosslinks, right for C-crosslinks) for all six pairs of overlapping triple helices present in the fibril (with one of the chains shown above the plot aligned to the x-axis). Moving average and standard errors for each curve were calculated over 100 ns simulation using period of 10 ns. **e**, Bond dissociation energy of carbon-carbon- (black) and carbon-nitrogen bond (gray) in collagen. Relative CASPT2 energies plotted against bond length extension.

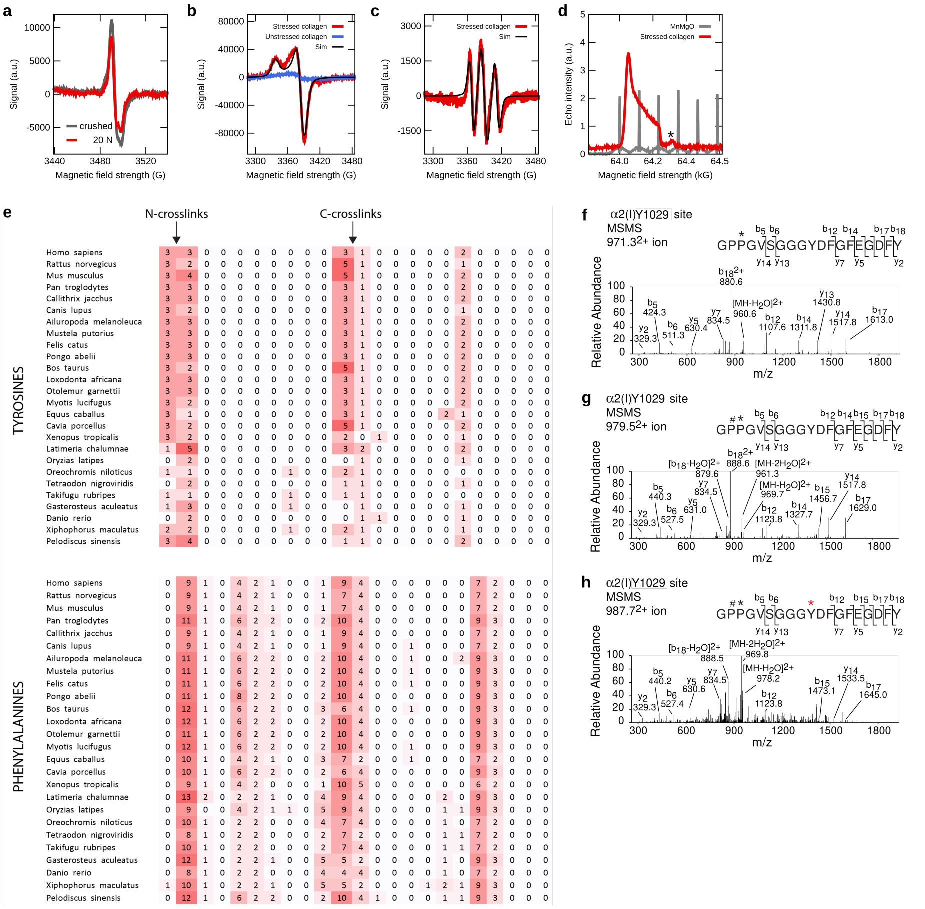

Supplementary Figure 3: **a**, Comparison of EPR signals at room temperature of a sample crushed with the mortar for 5 min (gray) and a sample pulled with 20 N using the LEX810 (red). The EPR signals are indistinguishable, showing that chemically same molecular scission is at play when pulling and crushing. **b**, Signal against magnetic field strength for a tendon crushed in liquid nitrogen and measured at 77 K with a microwave attenuation of 6 dB is in line with peroxy radical (Supplementary Table 1). The spectrum of an unstressed tendon at 77 K and 6 dB is shown in blue for reference. **c**, Subtraction of two spectra from the same experiment as in **b**, 30 dB and 9 dB, signal against magnetic field strength suggest a minor contribution from a methylene (CH_2^*) radical (Supplementary Table 1). **d**, Stressed collagen absorption signal (red) is plotted against the absolute magnetic field. To control the linearity and to determine the absolute magnetic field, MnO in MgO was measured under the same conditions and plotted with a scale factor of 0.1 in gray. The signal marked by * arises from the quartz capillary. **e**, Counts of Tyr and Phe residues for a given species calculated along the fibril from alignments of representative collagen type I sequences (see Methods). The counts were calculated using a moving average of 10 residues from each chain aligned across the fibril. **f-h**, MSMS spectra of the three different peptides shown in Fig. 3g in the main text. The predominant peptide (971.3 2+) contains a single 4-hydroxyproline (4Hyp). The peptide (979.5 2+) contains 4Hyp plus a 3-hydroxyproline (3Hyp) and the potential DOPA residue (Y1029) is found in peptide (987.7 2+).

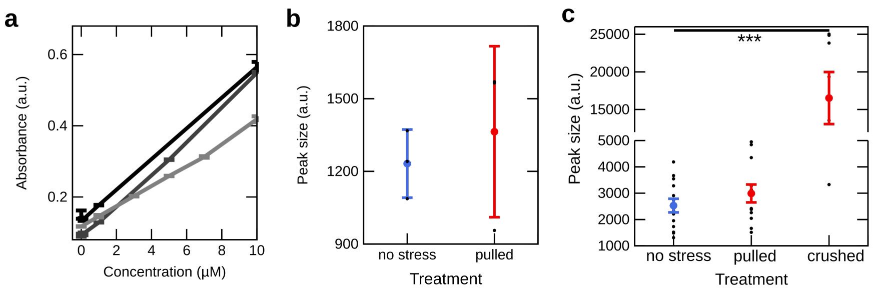

Supplementary Figure 4: **a**, H_2O_2 standard curve of the FOX assay, absorbance at 595 nm for different H_2O_2 concentrations. **b**, EPR peak size measured at room temperature for tendons kept at 50 %hum, in blue unstressed samples and in red samples pulled with a force of 15 N. Blue and red points show the mean and error bars the standard deviation, calculated from 3 independent EPR spectra, shown as black dots. **c**, EPR peak size of 1-Hydroxy-3-methoxycarbonyl-2,2,5,5-tetramethylpyrrolidine (CMH) supernatant of fascicles under different conditions. Blue and red points show the fascicles mass weighted mean and error bars the standard error of the mean. Both are calculated from 13 independent unstressed, 12 independent pulled with 15 N and 7 independent crushed fascicles, shown as black dots, respectively. Stars indicate two-tailed t-test p-values for samples with equal variance, $*** : p < 0.001$. Differences to the unstressed samples are significant only for the crushed not the pulled fascicles.

Supplementary Table 1: EPR parameters for the DOPA, the peroxy and the methylene radical from X-band (X) and G-band (G) measurements with systematical experimental error at different temperatures and quantum mechanical calculations (QM). DOPA* refers to the radical anion, DOPA-H* to the protonated and neutral radical. Line width and shape values were obtained from X-band or G-band EPR simulations. Line widths are given in Gauss, absolute values of isotropic hyperfine parameters a_{H} are given in Megahertz and temperatures in Kelvin. The number next to hydrogens indicates their position, see Fig. 3b. Line shape of simulated EPR spectra are defined proportionally by Lorentzian/Gaussian (L/G).

	DOPA* X	DOPA* G	DOPA* QM	DOPA-H* QM	Peroxy* X	Methylene* X
Temperature	300	40			77	77
g_x	2.0070	2.0071	2.0072	2.0091	2.0346	2.0025
	±0.00029	±0.00006			±0.00029	±0.00029
g_y	2.0050	2.0066	2.0064	2.0051	2.0065	2.0023
	±0.00029	±0.00006			±0.00029	±0.00029
g_z	2.0015	2.0022	2.0022	2.0022	2.0013	2.0020
	±0.00029	±0.00006			±0.00029	±0.00029
$a_{\mathrm{H}_{\beta 1}}$			0.9797	30.7356		70.00
$a_{\mathrm{H}_{\beta 2}}$			5.1027	34.2450		54.18
a_{H_2}			7.2970	6.5094		
a_{H_5}			8.2204	16.7114		
a_{H_6}			7.2048	4.2148		
Line width,						
x,y,z	3.6, 3.2, 3.0	20.0			12.6, 11.0, 12.0	6.0, 9.0, 9.0
Line shape	$L/G = 1.0$	$L/G = 1.0$			$L/G = 0.85$	$L/G = 0.8$

Supplementary Table 2: Diagonal hyperfine tensors, complementary to isotropic constants a_{iso} in Supplementary Table 1, $a_{x,y,z}$ are given in Megahertz.

		a_{x}	a_{y}	a_{z}	a_{iso}
DOPA* QM	$H_{\beta 1}$	3.08	−0.28	0.14	0.98
	$H_{\beta 2}$	7.83	3.80	3.68	5.10
	H_2	−12.35	−8.81	−0.73	−7.30
	H_5	−12.64	−10.30	−1.72	−8.22
	H_6	−11.08	−9.28	−1.25	−7.21
DOPA-H* QM	$H_{\beta 1}$	34.04	29.42	28.76	30.74
	$H_{\beta 2}$	37.56	32.99	32.19	34.25
	H_2	10.51	5.36	3.66	6.51
	H_5	−24.95	−19.00	−6.19	−16.71
	H_6	6.94	4.11	1.59	4.22
Methylene* X	H_1	61.65	72.86	78.47	70.00
	H_2	50.44	56.05	56.05	54.18
