# Supplementary material for: Mechanoradicals in tensed tendon collagen as a source of oxidative stress
Source: Nat Commun. 2020 May 8;11:2315. doi: 10.1038/s41467-020-15567-4 (PMC7210969; doi:10.1038/s41467-020-15567-4)
Supplement: Supplementary file 3 — Reporting Summary [file 41467_2020_15567_MOESM3_ESM.pdf]

nature research

Corresponding author(s): Dr. Frauke Gräter

Last updated by author(s): Feb 26, 2020

Reporting Summary

Nature Research wishes to improve the reproducibility of the work that we publish. This form provides structure for consistency and transparency in reporting. For further information on Nature Research policies, seeAuthors & Referees and theEditorial Policy Checklist .

Statistics

For all statistical analyses, confirm that the following items are present in the figure legend, table legend, main text, or Methods section.

n/a	Confirmed	
☐	☒	The exact sample size (*n*) for each experimental group/condition, given as a discrete number and unit of measurement
☐	☒	A statement on whether measurements were taken from distinct samples or whether the same sample was measured repeatedly
☐	☒	The statistical test(s) used AND whether they are one- or two-sided<br>*Only common tests should be described solely by name; describe more complex techniques in the Methods section.*
☒	☐	A description of all covariates tested
☒	☐	A description of any assumptions or corrections, such as tests of normality and adjustment for multiple comparisons
☐	☒	A full description of the statistical parameters including central tendency (e.g. means) or other basic estimates (e.g. regression coefficient) AND variation (e.g. standard deviation) or associated estimates of uncertainty (e.g. confidence intervals)
☒	☐	For null hypothesis testing, the test statistic (e.g. *F*, *t*, *r*) with confidence intervals, effect sizes, degrees of freedom and *P* value noted<br>*Give P values as exact values whenever suitable.*
☒	☐	For Bayesian analysis, information on the choice of priors and Markov chain Monte Carlo settings
☒	☐	For hierarchical and complex designs, identification of the appropriate level for tests and full reporting of outcomes
☒	☐	Estimates of effect sizes (e.g. Cohen's *d*, Pearson's *r*), indicating how they were calculated

Our web collection on statistics for biologists contains articles on many of the points above.

Software and code

Policy information about availability of computer code

Data collection	Gromacs version 2018, Gaussian version 09, Force Distribution Analysis for Gromacs version 2016.4, g_medeia, WinEPR, UvWin 3.00.000, THeBuScr, Modeller, CLANS, MAFFT, Molcas 8, ORCA 4.0, ImageJ
Data analysis	Python, Gnuplot, Origin, SimFiona, Easyspin, Thermo Xcalibur

For manuscripts utilizing custom algorithms or software that are central to the research but not yet described in published literature, software must be made available to editors/reviewers. We strongly encourage code deposition in a community repository (e.g. GitHub). See the Nature Research guidelines for submitting code & software for further information.

Data

Policy information about availability of data

All manuscripts must include a data availability statement. This statement should provide the following information, where applicable:

- Accession codes, unique identifiers, or web links for publicly available datasets
- A list of figures that have associated raw data
- A description of any restrictions on data availability

Data shown in all Figures 1-4 of the main text and all SI Figures is made accessible in the accompanying source data files. The atomistic collagen fibril model is part of the supplementary information. Full Molecular Dynamics trajectories can be made available any time upon request.

Field-specific reporting

Please select the one below that is the best fit for your research. If you are not sure, read the appropriate sections before making your selection.

Life sciences study design

All studies must disclose on these points even when the disclosure is negative.

Sample size	roughly 2 cm. Weight was determined by fine scale.
Data exclusions	none
Replication	varies between different experiments
Randomization	Tendon samples were allocated fully randomly.
Blinding	Tendon samples pulled at different forces were not blinded when collecting EPR or fluorometric data, but loading was directly followed by measuring each freshly pulled sample by the same investigator. Data collection (EPR or fluorescence) was unambiguous and quantitative, and not data was excluded (see above).

Reporting for specific materials, systems and methods

We require information from authors about some types of materials, experimental systems and methods used in many studies. Here, indicate whether each material, system or method listed is relevant to your study. If you are not sure if a list item applies to your research, read the appropriate section before selecting a response.

Materials & experimental systems

n/a	Involved in the study	
☒	☐	Antibodies
☒	☐	Eukaryotic cell lines
☒	☐	Palaeontology
☐	☒	Animals and other organisms
☒	☐	Human research participants
☒	☐	Clinical data

Methods

n/a	Involved in the study	
☒	☐	ChIP-seq
☒	☐	Flow cytometry
☒	☐	MRI-based neuroimaging

Animals and other organisms

Policy information about studies involving animals; ARRIVE guidelines recommended for reporting animal research

Laboratory animals	rattus norvegicus, spraque darley, male, nine weeks old.
Wild animals	The study did not include wild animals.
Field-collected samples	The study did not include samples collected from the field.
Ethics oversight	No ethical approval or guidance was required, because rat tails were harvested from dead animals and donated to us.

Note that full information on the approval of the study protocol must also be provided in the manuscript.
