# Supplementary figures and images for: Mechanoradicals in tensed tendon collagen as a source of oxidative stress
Source: Nat Commun. 2020 May 8;11:2315. doi: 10.1038/s41467-020-15567-4 (PMC7210969; doi:10.1038/s41467-020-15567-4)

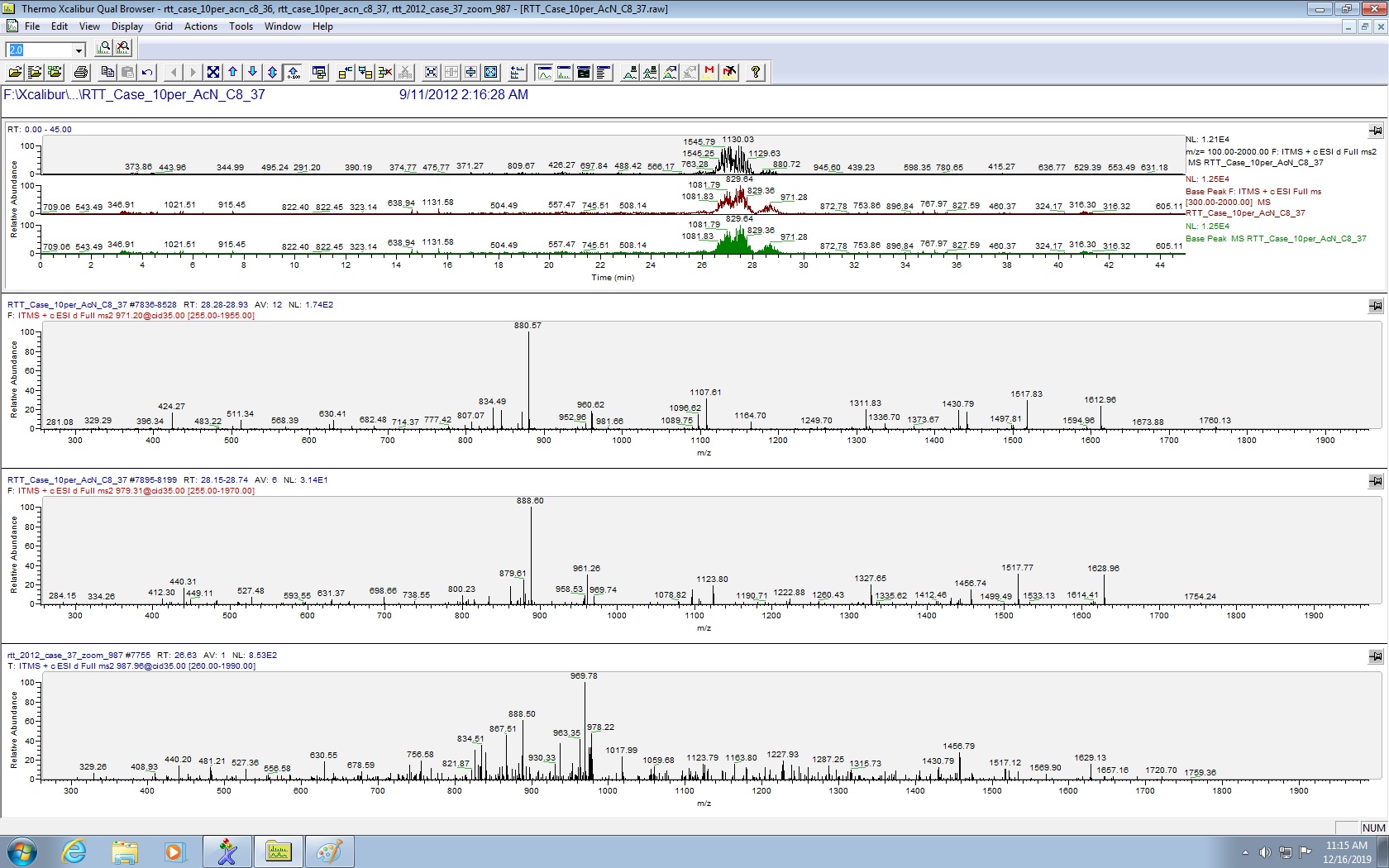

Supplement: Supplementary file 4 — Source Data [file 41467_2020_15567_MOESM4_ESM.zip › Fig_S3f-h.jpg]
